# Assessing of the relationship between renal function tests and retinopathy stage in patients with type II diabetes

**DOI:** 10.12861/jrip.2015.04

**Published:** 2015-03-01

**Authors:** Mohammad-Reza Tamadon, Raheb Ghorbani, Samaneh Rezaei, Gholamreza Daraei

**Affiliations:** ^1^Department of Internal medicine, Semnan University of Medical Sciences, Semnan, Iran; ^2^Research Center for Social Determinants of Health , Department of Community Medicine, Faculty of Medicine, Semnan University of Medical Sciences, Semnan, Iran; ^3^Department of Ophthalmology, Semnan University of Medical Sciences, Semnan, Iran

**Keywords:** Diabetic retinopathy, Nephropathy, Diabetes complications

## Abstract

**Introduction:** Retinopathy and nephropathy are long-term diabetes complications which are associated together. Renal dysfunction is a risk factor for progression and deterioration of diabetic retinopathy. Diabetes causes damage to the small blood vessels in the retina and kidney which eventually resulted in diabetic nephropathy, renal failure and blindness. Due to the high cost for treating of these complications it is better to prevent them.

**Objectives:** We aimed to assess the patients’ kidney function and retinal status in a group of diabetic patients to find probable association between nephropathy and retinopathy hence can prevent from serious renal complications.

**Patients and Methods:** In this cross-sectional study 253 patients with type 2 diabetes referring to ophthalmology clinics were evaluated. Eye examination was conducted by an ophthalmologist (vitreoretinal subspecialist) and disease stage was determined, then serum blood urea nitrogen (BUN) and creatinine tests and 24-hour urine collection for microalbuminuria were measured.

**Results:** Mean of BUN and microalbuminuria had significant difference in three groups including proliferative retinopathy, non-proliferative retinopathy and patients without retinopathy. The mean (± SD) of serum creatinine in patients with proliferative retinopathy, non-proliferative retinopathy and patients without retinopathy had no significant difference.

**Conclusion:** The presence or absence of retinopathy in the early stages of diabetic kidney disease has not related to renal involvement, in fact, patients without retinopathy may have renal involvement. In periodic examination, diabetic patients should be evaluated for microalbuminuria in addition to renal function test examination.

***Implication for health policy/practice/research/medical education *:**

Nephropathy and retinopathy is associated together so we can anticipate renal function status and its prognosis based on retina examination. In periodic examination, diabetic patients should be evaluated for microalbuminuria in addition to renal function test examination.

## Introduction

Diabetes mellitus has two types 1 and 2. Diabetes type 2 is a state which presents with different degrees of resistance to insulin and glucose production. The main cause of creating hyperglycemia characteristics in type 2 diabetes are genetic and metabolic defects in the performance or secretion of insulin (1).

Chronic complications of diabetes mellitus affect many organs and are responsible for the majority of complications and mortality caused by disease. Chronic complications divided into two groups: vascular and non-vascular. Vascular complications include retinopathy, nephropathy and neuropathy which usually appear two decades after of presence of hyperglycemia so in many people at time of diagnosis have these complications (1).

Diabetic retinopathy is the most important causes of blindness in the developed countries and is divided into two non-proliferative and proliferative stages. Diabetic non-proliferative retinopathy appears in the late first or early second decade of disease and the first pathologic changes include thickening of the endothelial basement membrane and reduce the pericytes number and capillary microaneurysms. Diabetic retinopathy presented as new vessels formation in retina and optic disc which proliferated into vitreous and eventually increases fibrous tissue, vitreous hemorrhage and tractional retinal detachment (2,3).

Diabetic nephropathy is a renal disease secondary to diabetes. Microalbuminuria (30-300 milligrams of albumin in 24-hour urine collection) increased the risk of diabetic nephropathy progression up to 10 to 20 times. Even in presence of microalbuminuria, occurrence of renal failure could be prevented or even completely treated by control of blood glucose level and blood pressure (4). Retinopathy and nephropathy associated together and renal malfunction in diabetics is a risk factor for progression and deterioration of diabetic retinopathy (5).

## Objectives

With regard to the impact of diabetes on small vessels in retina and kidney as well as creation of diabetic nephropathy, retinopathy and eventually blindness and renal failure, we aimed to anticipate the renal function and its prognosis based on retina status prediction and provide appropriate management for prevention of serious renal complications.

## Patients and Methods

### Patients

In this study cross-sectional, from all patients with type 2 diabetes who referred to Amir-Al-Momenin ophthalmology clinic in Semnan, for periodic examinations from March 2011 to February 2012, 256 patients enrolled after written consent regardless of diabetes duration. Patients examined by ophthalmologist and stage of disease were determined based on diabetic retinopathy study (DRS). Laboratory tests including blood urea nitrogen (BUN), serum creatinine level and albumin were done in 24-hour urine collection.

### Ethical issues

1. The research followed the tenets of the Declaration of Helsinki; 2. Informed consent was obtained; and 3. The research was approved by the ethical committee of Semnan University of Medical Sciences.

### Data analysis

Demographic data as well as results of the tests and ophthalmologic examinations were recorded. For univariate analysis, we used Kolmogorov–Smirnov, Chi-square, one-way variance analysis and Tukey tests. In a multivariate analysis, we used stepwise logistic regression analysis assigning creatinine (abnormal= 1, normal= 0) as the dependent factor and the other associated variables as independent components. We used SPSS version 16.00 (SPSS, Inc., Chicago, IL). The P value less than 0.05 were considered statistically significant.

## Results

Mean ± Standard deviation of age of patients with proliferative, non-proliferative and without retinopathy was 56.9 ± 11.5, 59.3± 9.4 and 59.0 ± 7.9 years respectively which difference was not significant (P= 0.221). 48.2% of patients with proliferative retinopathy, 61.2% with non-proliferative retinopathy and 62.4% of patients without retinopathy were female. Gender distribution had not significant difference in three groups (P= 0.121;  Table 1). Mean ± SD of urine albumin level in patients with proliferative retinopathy, non-proliferative retinopathy and without retinopathy was 19.8 ± 6.6, 18.5 ± 6.7 and 16.8 ± 6.3 mg/day respectively that the difference was significant (P= 0.012). Mean (±SD) of albumin in patients with proliferative retinopathy was significantly more than it in patients without retinopathy (P= 0.009;  Table 2).

**Table 1:** Age distribution of diabetic patients based on retinopathy stages

**Variables**		**Retinopathy stages**					
		**Without retinopathy**		**Non proliferative retinopathy**		**Proliferative retinopathy**	
		**No.**	**%**	**No.**	**%**	**No.**	**%**
Age groups	< 50	17	20.0	9	10.6	3	3.6
	50-59	35	41.2	37	34.5	45	54.2
	60-69	23	27.1	25	29.4	25	30.1
	≤ 70	10	11.8	14	16.5	10	12.0
Gender	Male	53	62.4	52	61.2	40	48.2
	Female	32	37.6	33	38.8	43	51.8

**Table 2:** Presence of albuminuria based on retinopathy stages

**Albuminuria**	**Retinopathy stages**					
	**Without retinopathy**		**Non proliferative retinopathy**		**Proliferative retinopathy**	
	**No.**	**%**	**No.**	**%**	**No.**	**%**
+	4	4.7	3	3.5	3	3.6
**-**	81	95.3	82	96.5	80	96.4
Total	100	85		85	100	83

Mean ± SD of BUN in patients with proliferative retinopathy, non-proliferative retinopathy and patients without retinopathy was 18.9 ± 7.8, 17.6 ± 4.3 and 16.3 ± 2.5 mg/dl respectively, while the differences was significant (P= 0.008). The BUN level in patients with proliferative retinopathy was significantly more than it in patients without retinopathy (P= 0.005). Mean ± SD of serum creatinine in patients with proliferative retinopathy, non-proliferative retinopathy and patients without retinopathy was 1.13 ± 0.43, 1.17 ± 0.95 and 0.98 ± 0.17 respectively which the difference was not significant (P= 0.107;  Table 3).

**Table 3:** Mean ± SD of BUN and creatinine in diabetic patients based on retinopathy stages

**Parameters**	**Stages of retinopathy**	**Number**	**Mean**	**Standard deviation**	**P value**
BUN					
	PR	83	18.9	7.8	0.008
	NPR	85	17.5	4.3	
	WR	85	16.3	2.5	
Creatinine					
	PR	83	1.13	0.43	0.107
	NPR	85	1.17	0.95	
	WR	85	0.98	0.17	

However, 78.3% of patients with proliferative retinopathy, 72.9% with non-proliferative retinopathy and 90.6% of patients without retinopathy had normal creatinine levels which difference between patients with non-proliferative retinopathy and patients without retinopathy was significant (P= 0.023), so after age and sex adjustment, chance for abnormal creatinine in patients with non-proliferative retinopathy was 3.42 times more than that of patients without retinopathy (OR = 3.42, 95% CI: 1.42- 8.27, P= 0.009) and 2.60 times in patients with proliferative retinopathy in compared with patients without retinopathy (OR = 2.60, 95% CI: 1.05-6.42, P= 0.039).

## Discussion

In previous studies there was less attention to renal function tests in diabetics with proliferative, and non-proliferative and without retinopathy. In a study conducted by Tam *et al*. in Hong Kong, renal function tests did not evaluate in various types of diabetic retinopathy, however in our study we assessed three groups including patients with proliferative retinopathy, non-proliferative retinopathy and without retinopathy separately (6). In a study conducted by Boeller *et al.*, only microalbuminuria was evaluated in these patients but in our study, BUN and creatinine were also evaluated in patients (7). Also, in a study conducted by Asensio-Sanchez et al. in Spain, percentage of microalbuminuria was not calculated in diabetic patients but we accurately calculated mean and percentage of microalbuminuria in the three groups (8).

The results of a study which was conducted by the Cia *et al.* in China (9), are similar to our study for BUN and microalbuminuria but creatinine had not any significant difference between the three groups and it can be explained that the normal ophthalmology examination in the early stages did not represent lower renal involvement proportionally so that the patients without retinopathy may have renal involvement.

## Conclusion

Nephropathy and retinopathy are associated together so we can anticipate renal function status and its prognosis based on retina examination. But this does not mean that patients without retinopathy have not renal involvement. In fact, diabetic patients without retinopathy may partially have microalbuminuria which is due to renal involvement resulted from diabetes.

## Authors’ contributions

All authors wrote the paper equally.

## Conflict of interests

The authors declared no competing interests.

## Ethical considerations

Ethical issues (including plagiarism, misconduct, data fabrication, falsification, double publication or submission, redundancy) have been completely observed

by the authors.

## Funding/Support

This article was extracted from thesis by Samaneh Rezaei for M.D. degree. This study was supported by a grant from Semnan University of Medical Sciences (grant No. UN122).
